# Treatment of Cancer by Bromine

**Published:** 1878-10

**Authors:** 


Treatment of Cancer by Bromine.—Dr. Novaro, in the *Giornale della Academia de Foirino*, speaks of scarification and the cauterization of cancer by bromine, as much more successful than any other treatment. As results he states that—

1. It produces a temporary, if not a permanent, cure of cancerous tumors, with less loss of substance than the knife or actual cautery.

2. This treatment can be applied to the neck of the uterus better than the knife or burning iron.

3. That scarification produces but little hemorrhage.

4. That the peritoneum can be touched without fear of grave inflammation, in which Simon coincides.

